# Mitochondrial DNA haplogroup M7 confers a reduced risk of colorectal cancer in a Han population from northern China

**DOI:** 10.1111/jcmm.16789

**Published:** 2021-07-19

**Authors:** Qing Yuan, Liping Su, Tian Wang, Yang Liu, Zhenxing Lu, Kaixiang Zhou, Shanshan Guo, Xiwen Gu, Jinliang Xing, Xu Guo

**Affiliations:** ^1^ Institute of Medical Research Northwestern Polytechnical University Xi’an China; ^2^ State Key Laboratory of Cancer Biology and Department of Physiology and Pathophysiology Fourth Military Medical University Xi’an China; ^3^ Laboratory Department Shaanxi Provincial People's Hospital Xi’an China; ^4^ Key Laboratory of Shaanxi Province for Craniofacial Precision Medicine Research Clinical Research Center of Shaanxi Province for Dental and Maxillofacial Diseases College of Stomatology Xi'an Jiaotong University Xi'an China

**Keywords:** colorectal cancer risk, haplogroup, mitochondria, single nucleotide polymorphisms

## Abstract

Mitochondria are central eukaryotic organelles in cellular metabolism and ATP production. Mitochondrial DNA (mtDNA) alterations have been implicated in the development of colorectal cancer (CRC). However, there are few reports on the association between mtDNA haplogroups or single nucleotide polymorphisms (SNPs) and the risk of CRC. The mtDNA of 286 Northern Han Chinese CRC patients were sequenced by next‐generation sequencing technology. MtDNA data from 811 Han Chinese population controls were collected from two public data sets. Then, logistic regression analysis was used to determine the effect of mtDNA haplogroup or SNP on the risk of CRC. We found that patients with haplogroup M7 exhibited a reduced risk of CRC when compared to patients with other haplogroups (odds ratio [OR] = 0.532, 95% confidence interval [CI] = 0.285–0.937, *p* = 0.036) or haplogroup B (OR = 0.477, 95% CI = 0.238–0.916, *p* = 0.030). Furthermore, haplogroup M7 was still associated with the risk of CRC when the validation and combined control cohort were used. In addition, several haplogroup M7 specific SNPs, including 199T>C, 4071C>T and 6455C>T, were significantly associated with the risk of CRC. Our results indicate the risk potential of mtDNA haplogroup M7 and SNPs in CRC in Northern China.

## 1 INTRODUCTION

Colorectal cancer (CRC) is one of the most common cancers in both men and women, accounting for nearly 10% of the global cancer incidence.^1^ Approximately 274,800 new CRC cases and 132,100 CRC‐related deaths have been estimated to occur in China each year, accounting for nearly one‐tenth of the global CRC burden.^2^ Assessing the risk of CRC and then screening is the most powerful public health tool to reduce mortality or incidence.^3^, ^4^ The major risk factors that may influence the development of the CRC include age, male gender, obesity, diet high in fat as well as the medical history of inflammatory bowel disease (IBD), diabetes mellitus and so on.^5^ Several prediction models have been developed to quantify CRC risk based on clinical or laboratory data. However, all these models still have obvious limitations, such as the restricted age range of subjects, selection bias of subjects and incomplete assessment of risk factors in these studies.^5^, ^6^, ^7^ Meanwhile, the diagnosis of CRC mainly depends on imaging techniques, which are less efficient for the early detection of CRC. Accurate early detection or risk prediction contributes to improved CRC survival. Unfortunately, to date, there are no favourable biomarkers for the risk prediction of CRC.

Intensive efforts have been made to understand the genetic risk factors of CRC. To date, over 40 nuclear genome variants associated with CRC risk have been identified, including SNP rs10911251, rs1321311, rs1035209 and so on.^8^, ^9^, ^10^ However, these susceptibility loci account for only about 8%–16% of CRC cases, suggesting that additional genetic risk factors of CRC may remain to be explored.^8^

Mitochondria are central eukaryotic organelles in cellular metabolism and ATP production. Notably, multiple metabolic deregulations have been linked to the pathogenesis of CRC, including amino acid metabolism, glucose metabolism and lipid metabolism.^11^ Somatic mtDNA mutations and copy number alterations have also been frequently observed in CRC samples.^12^ However, the potential involvement of germline mtDNA variations in CRC development is less known.

Germline mtDNA variations are often characterized by assigning haplogroups, which can be defined by a certain set of mtDNA variants and reflect specific ancestral populations and geographic origins. At present, it has been proved that mtDNA haplogroups are associated with the risk of various cancers.^13^, ^14^, ^15^, ^16^ Grzybowski et al. found that haplogroup R and its diagnostic mutations at positions 12705 and 16223 are associated with higher frequencies in Polish CRC patients when compared to healthy individuals.^17^ Certain mtDNA SNPs have also been associated with an increased risk of CRC in Iranian or European Americans.^18^, ^19^, ^20^, ^21^, ^22^ However, mtDNA haplogroups are highly variable among ethnic populations originating from different geographical locations. It is still unclear whether mtDNA haplogroups or SNPs are an important risk factor for colorectal carcinogenesis in the Han Chinese population.

To assess the contribution of mtDNA haplogroups and SNPs to the prevalence of CRC, we conducted a case‐control study in a Han Chinese population.

## 2 MATERIALS AND METHODS

### 2.1 Patient specimens and DNA extraction

A total of 286 CRC patients were enrolled from the Tangdu Hospital affiliated with the Fourth Military Medical University (FMMU) (China) from January 2008 to December 2014. The diagnosis of CRC was confirmed by histological pathology. Patients’ demographic and clinical data, including age, gender, tumour position, degree of differentiation, tumour node metastasis (TNM) stage and carcinoembryonic antigen (CEA), were collected and analysed. Tumour and paired non‐tumour tissue specimens were collected from each patient during the surgical operation. Haematoxylin‐eosin‐stained slides of CRC tumour tissues were carefully reviewed to confirm that the cancer cell content was more than 90% in tumour tissues. Then, Genomic DNA was extracted from tissues of CRC patients who were enrolled in our previous study,^23^ according to the E.N.Z.A. Tissue DNA Kit (Omega Bio‐Tek, Inc., Doraville, GA) under the manufacturer's instructions. DNA quantity and quality were analysed by Qubit (Invitrogen) and the 2100 Bioanalyzer system (Agilent Technologies), respectively. The study was approved by the Ethics Committees of the FMMU and written informed consent was obtained from each patient.

### 2.2 Capture‐based mtDNA next‐generation sequencing

To obtain the full spectrum of germline mtDNA variations in CRC patients, we performed capture‐based mtDNA next‐generation sequencing as previously described.^24^ Briefly, genomic DNA (1 μg for each sample) was randomly sonicated by Sonicator (Scientz98) to obtain fragments mainly distributed at 300–500 bp. The sonicated DNA fragments were end‐repaired, ligated with sequencing adapters and amplified to generate the whole genome sequencing (WGS) library. Then, the WGS libraries of 20 samples were mixed with homemade biotinylated mtDNA capture probes for hybridization. After PCR amplification and purification, the mtDNA capture quality was determined using agarose gel electrophoresis and real‐time fluorescence quantification. Finally, the captured mtDNA libraries were sequenced on an Illumina X Ten platform using paired‐end runs with 2 × 150 cycles (PE 150).

### 2.3 mtDNA haplogroup and mtSNPs

The FASTQ preprocessor fastp software (version 0.20.0)^25^ was used to perform quality control and adapter trimming on the raw sequencing data. BWA software (version 0.7.17‐r1188) was used to map the trimmed reads. To minimize the contamination of nuclear mtDNA segments, trimmed reads were mapped to the Revised Cambridge Reference Sequence (rCRS) of mtDNA and the reference genome (hg19). Next, Picard tools (version 1.81) were used to mark and remove duplicate reads. To reduce the false‐positive rate of nearby indel positions, local realignment was performed using IndelRealigner in GATK software (version 3.2–2). Then, mtDNA sequences were extracted into FASTA format with the Perl script written in our laboratory. The obtained FASTA sequences were analysed using MitoTool (www.mitotool.org) ^26^ to determine mtDNA haplogroups and SNPs. Macro‐haplogroups and micro‐haplogroups were annotated based on the PhyloTree (www.phylotree.org). ^27^ The variation which was observed in both tumour and paired non‐tumour tissues was defined as mtDNA SNP. SNPs with minor allele frequency (MAF) <5% in cases and controls were excluded in further analysis.

### 2.4 Control cohorts

Two independent Han Chinese cohorts were used to evaluate the risk of CRC. First, we collected information on mtDNA haplogroups and SNPs from a published data set, which included 562 normal individuals in Shaanxi Province of Northern China.^28^ To further validate the results, we also collected another control cohort from the 1000 Genome Project,^29^ including 249 Chinese (Southern Han Chinese, Beijing Han Chinese from Northern China and Denver Han Chinese from Colorado).

### 2.5 Statistical analysis

In a case‐control study, haplogroups with frequency>1% in both the controls and CRC patients were analysed to evaluate the effect of common mtDNA haplogroup on CRC. To estimate the relative risk, odds ratios (ORs) and 95% confidence intervals (CIs) were calculated for each haplogroup and SNP. Logistic regression analysis was used to assess the associations between haplogroups or SNPs and the risk of CRC. *p*‐values of less than 0.05 were considered statistically significant. When other haplogroups were used as the reference haplogroup, it refers to the comparison of a specific haplogroup with all other haplogroups. Statistical analyses were performed using R software (version 4.0.2).

## 3 RESULTS

### 3.1 Patients characteristics

The clinical characteristics of the 286 CRC patients are summarized in Table 1. The median age of patients was 61 years old (ranging from 28–87 years) and 149 (52.10%) patients were male. Most CRC patients (93.71%) had CRC differentiation grade I‐II, 164 (57.34%) patients were diagnosed with TNM stage I‐II and 172 (60.14%) patients had serum CEA <5 ng/ml. The prevalence of major haplogroups is also listed in these different clinicopathological categories (Table S1).

**TABLE 1:** Clinical‐pathological characteristics of 286 Han Chinese CRC patients

Characteristics	CRC, *n* (%) *n* = 286
Age (years)	
≤61	141 (49.30%)
>61	145 (50.70%)
Gender	
Male	149 (52.10%)
Female	137 (47.90%)
Position	
Colon	194 (67.83%)
Rectum	92 (32.17%)
Differentiation	
I—II	268 (93.71%)
III	18 (6.29%)
TNM stage	
I—II	164 (57.34%)
III—IV	122 (42.66%)
Serum CEA (ng/ml)	
<5	172 (60.14%)
≥5	109 (38.11%)
Unknown	5 (1.75%)

Abbreviations: CEA, Carcinoembryonic antigenCRC, Colorectal cancer; TNM, Tumour node metastasis.

### 3.2 Association between mtDNA haplogroups and colorectal cancer risk in northern Han population

To investigate the association between genetic mtDNA variation and CRC risk, mtDNA haplogroups were annotated in 286 CRC cases and 562 Northern Han Chinese healthy controls (cohort 1). As shown in Table 2, patients were categorized into ten major haplogroups. Among them, haplogroup D was the most prevalent clade in both CRC patients and healthy controls (51 cases [17.83%] and 126 controls [22.42%]), followed by haplogroup B (51 cases [17.83%] and 86 controls [15.30%]).

**TABLE 2:** Association between mtDNA haplogroups and CRC risk with other haplogroups as reference group

Haplogroup	Case (*n* = 286)	Control (*n* = 562)	OR (95%CI)	*p*‐value
A	27 (9.44%)	56 (9.96%)	0.942 (0.574–1.513)	0.808
B	51 (17.83%)	86 (15.30%)	1.201 (0.818–1.751)	0.344
D	51 (17.83%)	126 (22.42%)	0.751 (0.520–1.073)	0.121
G	13 (4.55%)	30 (5.34%)	0.844 (0.420–1.611)	0.619
M7	15 (5.24%)	53 (9.43%)	**0.532 (0.285–0.937)**	**0.036**
M8	34 (11.89%)	59 (10.50%)	1.150 (0.729–1.791)	0.541
M9	7 (2.45%)	8 (1.42%)	1.737 (0.604–4.888)	0.291
M10	10 (3.50%)	10 (1.78%)	2.000 (0.812–4.929)	0.126
N9	22 (7.69%)	33 (5.87%)	1.336 (0.754–2.323)	0.310
R9	42 (14.69%)	81 (14.41%)	1.022 (0.678–1.522)	0.915
Others	14 (4.90%)	20 (3.56%)	1.395 (0.680–2.785)	0.350

Bold entries indicate statistical significance.

When compared with other haplogroups, haplogroup M7 had a much lower percentage in CRC patients (5.24%, *n* = 15) than in control cohort 1 (9.43%, *n* = 53), which corresponded to a significantly reduced risk of CRC (OR 0.532, [95% CI 0.285–0.937], *p* = 0.036) (Table 2). The reduced CRC risk in haplogroup M7 was also evident when haplogroup B was selected as the reference haplogroup (OR 0.477, [95% CI 0.238–0.916], *p* = 0.030) (Table 3). Although no significant association was found when haplogroup D was used as the reference haplogroup, the selection of haplogroup D as a reference may be inappropriate due to the apparent imbalance of haplogroup D between the CRC and control cohort (Table 2).

**TABLE 3:** Association between mtDNA haplogroups and CRC risk with haplogroup B and D as reference group

Haplogroup	Case (*n* = 286)	Control (*n* = 562)	OR(95%CI)	*p*‐value	OR(95%CI)	*p*‐value
B	51 (17.83%)	86 (15.30%)	Ref^a^		1.465 (0.911–2.360)	0.115
A	27 (9.44%)	56 (9.96%)	0.813 (0.454–1.439)	0.481	1.191 (0.674–2.082)	0.542
D	51 (17.83%)	126 (22.42%)	0.683 (0.424–1.098)	0.115	Ref^b^	
G	13 (4.55%)	30 (5.34%)	0.731 (0.341–1.503)	0.404	1.071 (0.504–2.180)	0.854
M7	15 (5.24%)	53 (9.43%)	**0.477 (0.238–0.916)**	**0.030**	0.699 (0.353–1.328)	0.287
M8	34 (11.89%)	59 (10.50%)	0.972 (0.561–1.675)	0.918	1.424 (0.833–2.423)	0.194
M9	7 (2.45%)	8 (1.42%)	1.475 (0.491–4.348)	0.477	2.162 (0.724–6.330)	0.156
M10	10 (3.50%)	10 (1.78%)	1.686 (0.650–4.381)	0.277	2.471 (0.959–6.373)	0.058
N9	22 (7.69%)	33 (5.87%)	1.124 (0.588–2.128)	0.720	1.647 (0.871–3.084)	0.121
R9	42 (14.69%)	81 (14.41%)	0.874 (0.524–1.453)	0.605	1.281 (0.780–2.101)	0.326
Others	14 (4.90%)	20 (3.56%)	1.180 (0.541–2.526)	0.671	1.729 (0.799–3.667)	0.156

^a^ Haplogroup B was used as reference group.

^b^ Haplogroup D was used as reference group.

Bold entries indicate statistical significance.

### 3.3 Association between haplogroup M7 and CRC risk with validation cohort

To further validate the association of haplogroup M7 with the risk of CRC, we used another Han Chinese data set from the 1000 genome project, which consisted of 249 individuals (Southern Han, 55; Northern Han, 121; and Denver Han, 73). As shown in Table 4, patients with haplogroup M7 were still associated with a significantly reduced risk of CRC when other haplogroups (OR 0.429, [95% CI 0.217–0.808], *p* = 0.011) and haplogroup B (OR 0.431, [95% CI 0.200–0.901], *p* = 0.028) were selected as the reference haplogroup. The association between haplogroup M7 and reduced CRC risk was also supported by a marginal significance level when haplogroup D was used as reference (OR 0.504, [95% CI 0.236–1.046], *p* = 0.070).

**TABLE 4:** Validation of association between mtDNA haplogroups and CRC risk

Haplogroup	Case(*n* = 286)	Validation control cohort (*n* = 249)	OR(95%CI)	*p*‐value	Combined control cohort (*n* = 811)	OR(95%CI)	*p*‐value
M7	15 (5.24%)	28 (11.24%)	**0.427 (0.217–0.808)**	**0.011** ^a^	81 (9.99%)	**0.499 (0.272–0.855)**	**0.017** ^a^
B	51 (17.83%)	41 (16.47%)	Ref	**0.028** ^b^	127 (15.66%)	Ref	**0.018** ^b^
M7	15 (5.24%)	28 (11.24%)	**0.431 (0.200–0.901)**		81 (9.99%)	**0.461 (0.237–0.857)**	
D	51 (17.83%)	48 (19.28%)	Ref	0.070^a^	174 (21.45%)	Ref	0.155^a^
M7	15 (5.24%)	28 (11.24%)	0.504 (0.236–1.046)		81 (9.99%)	0.632 (0.326–1.166)	

^a^ Other haplogroups were used as reference group.

^b^ Haplogroup B was used as reference group.

^c^ Haplogroup D was used as reference group.

Bold entries indicate statistical significance.

Based on the combined control cohort (cohort 1&2, *n* = 811), we also found that haplogroup M7 was a protective factor for the onset of CRC when referring to other haplogroups (OR 0.499, [95% CI 0.272–0.855], *p* = 0.017) and haplogroup B (OR 0.461, [95% CI 0.237–0.857], *p* = 0.018).

### 3.4 The association between mtDNA SNPs and CRC risk

To clarify the association between mtDNA SNPs and CRC risk, we screened for common SNPs with allele frequencies higher than 5% in our cohorts. Using this criterion, 53 SNPs were identified in control cohort 1 (Table S2), 72 SNPs were identified in control cohort 2 (Table S3) and 58 SNPs were identified in the combined control cohort (Table S4).

**FIGURE 1:**
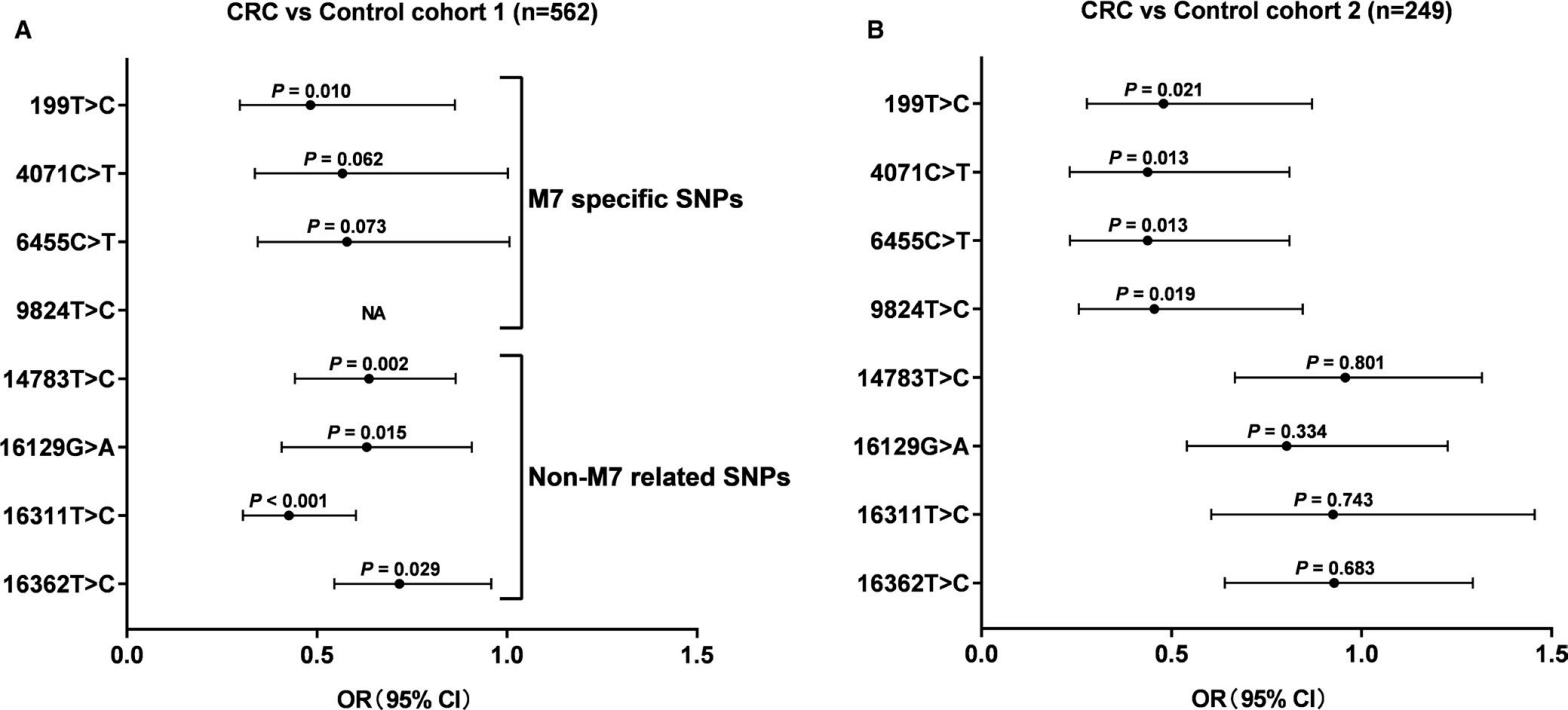
Association between mtDNA SNPs and the risk of CRC. (A) Using logistic regression to analyze the correlation between SNPs and CRC in control cohort 1 (*n* = 562). The abscissa represents the odds ratio (OR) value and 95% confidence interval (CI), and the ordinate represents different SNPs. (B) The correlation analysis between SNPs and CRC was performed in the method of logistic regression in validation control cohort (*n* = 249).

As shown in Figure 1, three haplogroup M7‐specific SNPs, including 199T>C, 4071C>T, and 6455C>T, exhibited a stable association with the reduced risk of CRC in both cohorts. Although SNP 4071C>T and 6455C>T did not significantly indicate a decreased risk of CRC in control cohort 1 (*p* = 0.062 and *p* = 0.073, Figure 1A), which may partially be due to sampling bias. However, these three haplogroup M7‐specific SNPs were significantly associated with the risk of CRC in control cohort 2 (Figure 1B). Another haplogroup M7 specific SNP 9824T>C also showed a significant association with CRC only in control cohort 2 (*p* = 0.019), because 9824T>C was not detected in control cohort 1.

Moreover, four SNPs catalogued to non‐M7 haplogroup, including 14783T>C, 16129G>A, 16311T>C and 16362T>C, also showed significant associations with CRC risk in control cohort1 (*p* = 0.002, *p* = 0.015, *p* < 0.001, *p* = 0.029), but these associations may be artificial due to the complete lack of support in control cohort 2. Thus, the association of haplogroup M7 specific SNPs with CRC provides further evidence that haplogroup M7 is associated with reduced CRC risk in the Han Chinese population.

## 4 DISCUSSION

In this study, we comprehensively analysed the association between the mtDNA haplogroups, SNPs and CRC risk in the Northern Han Chinese population. We sequenced mitochondrial genomes from 286 CRC patients and retrieved mtDNA haplogroup data form 562 and 249 Han Chinese normal cohorts from public data sets. We found that haplogroup M7 was significantly associated with a reduced risk of CRC.

Several studies have reported the association between mtDNA haplogroup M7 and disease risk in the Han Chinese population. For instance, haplogroup M7 has been reported as a risk factor for lung cancer (OR = 2.037, 95%CI = 1.253–3.312, *p* = 0.004)^30^ in the Southwestern Chinese population. For this study, haplogroup M7 was found to be present in cases at frequencies of 12.67% but was present in controls at frequencies of 5.95%. However, haplogroup M7 was present in controls from Northwestern Chinese at frequencies of 9.43% in our study, it suggested that the opposite associations of haplogroup M7 and diseases exist in different areas of China. We have previously found that haplogroup M7 was associated with a significant reduction in the risk of liver cancer in Northern China.^31^ More recently, Sun et al. reported that haplogroup M7 is associated with an increased risk of disability in an ageing Chinese population.^32^ In this study, we further found that haplogroup M7 was associated with a reduced risk of CRC. Thus, our results provide further evidence for the involvement of mtDNA haplogroup M7 in ageing‐related human diseases, especially cancer.

It is currently unclear that how mtDNA haplogroup M7 may affect the risk of CRC or other diseases. As a unique set of mtDNA polymorphisms, mtDNA haplogroups represent historical mutations accumulated by discrete maternal lineages.^33^ In this regard, haplogroup‐associated mtDNA variations have the potential to influence mitochondrial OXPHOS function and/or the generation of reactive oxygen species,^34^, ^35^ both of which may modify the risk of cancer. Interestingly, it has been reported that, compared to haplogroup M8, haplogroup M7 has a significantly lower respiratory chain activity and lower mitochondrial membrane potential, together with a 40% reduction in respiration‐related oxygen consumption. These findings hint that mitochondrial haplogroup M7 may reduce the risk of CRC due to the presence of sub‐optimal mitochondrial functionality in this haplogroup.^32^ Lower mitochondrial function may promote tumorigenesis due to altered mitochondrial signals including ROS and NAD^+^/NADH, which are responsible for overactivation of mitochondrial retrograde signaling.^36^ However, we do not know how cellular metabolism pathways are regulated by mitochondrial function in haplogroup M7 containing cells. Further studies on the causal relationship between OXPHOS function and cellular metabolism pathways are necessary to completely reveal the protective role of haplogroup M7 in CRC.

This study has some limitations. One of the limitations was the small sample size. As mitochondrial haplogroup M7 only accounted for 5.2% of our CRC patient cohort and 9.4% and 11.2% in the two control cohorts, it is necessary to validate our findings in a large, independent case‐control study. The control cohort 2 was consisted with Han samples from south and north China, which will also introduce some bias to the results as the higher distribution frequency of haplogroup M7 in south China. Furthermore, the association between mtDNA haplogroup M7 and CRC risk should be further validated by functional studies, which are greatly hindered by our inability to accurately manipulate the mitochondrial genome. In addition, since the majority of mitochondrial respiratory chain proteins are encoded in the nuclear genome, the association between haplogroup M7 and reduced CRC risk may also be affected by the genetic background of the host's nuclear genome.

In conclusion, our study analysed the relationship between mitochondrial genetic variations and the risk of CRC. It revealed for the first time that mtDNA haplogroup M7 reduced the risk of CRC in the Northern Han Chinese population. Our study points to the importance of the mitochondrial genetic background in the development of colorectal cancer.

## CONFLICT OF INTEREST

The authors declare that they have no competing interests.

## AUTHOR CONTRIBUTION

**Qing Yuan:** Software (lead); Visualization (lead); Writing‐original draft (lead). **Liping Su:** Writing‐original draft (equal). **Tian Wang:** Writing‐original draft (supporting). **Yang Liu:** Project administration (equal). **Zhenxing Lu:** Formal analysis (equal). **Kaixiang Zhou:** Project administration (equal). **Shanshan Guo:** Formal analysis (equal). **Xiwen Gu:** Writing‐review & editing (supporting). **Jinliang Xing:** Writing‐review & editing (equal). **Xu Guo:** Writing‐review & editing (lead).

## Supporting information

Supplementary Material
Supplement

## Data Availability

The data that supports the findings of this study are available in the supplementary material of this article.
