# Supplementary material for: Mitochondrial DNA haplogroup M7 confers a reduced risk of colorectal cancer in a Han population from northern China
Source: J Cell Mol Med. 2021 Jul 19;25(15):7538–44. doi: 10.1111/jcmm.16789 (PMC8335663; doi:10.1111/jcmm.16789)
Supplement: Supplementary file 1 — Supplementary Material Supplement [file JCMM-25-7538-s001.docx]

**Supplementary**

**Table S1. Clinical-pathological characteristics of 286 Han Chinese CRC patients in different haplogroup**

| **Characteristics** | **CRC, n (%) n=286** | **Haplogroup** | | | | | | | | | | | |
| --- | --- | --- | --- | --- | --- | --- | --- | --- | --- | --- | --- | --- | --- |
|  |  | **A** | **B** | **D** | **G** | **M7** | **M8** | **M9** | **M10** | **N9** | **R9** | **Others** |  |
| **Age (years)** |  | **27** | **51** | **51** | **13** | **15** | **34** | **7** | **10** | **22** | **42** | **14** |  |
| ≤ 61 | 141 (49.30%) | 12 | 26 | 19 | 10 | 7 | 16 | 6 | 6 | 13 | 22 | 4 |  |
| > 61 | 145 (50.70%) | 15 | 25 | 32 | 3 | 8 | 18 | 1 | 4 | 9 | 20 | 10 |  |
| **Gender** |  |  |  |  |  |  |  |  |  |  |  |  |  |
| Male | 149 (52.10%) | 14 | 27 | 28 | 8 | 7 | 16 | 0 | 4 | 12 | 25 | 8 |  |
| Female | 137 (47.90%) | 13 | 24 | 23 | 5 | 8 | 18 | 7 | 6 | 10 | 17 | 6 |  |
| **Position** |  |  |  |  |  |  |  |  |  |  |  |  |  |
| Colon | 194 (67.83%) | 16 | 32 | 36 | 9 | 10 | 22 | 5 | 8 | 16 | 30 | 10 |  |
| Rectum | 92 (32.17%) | 11 | 19 | 15 | 4 | 5 | 12 | 2 | 2 | 6 | 12 | 4 |  |
| **Differentiation** |  |  |  |  |  |  |  |  |  |  |  |  |  |
| I—II | 268 (93.71%) | 24 | 50 | 49 | 13 | 15 | 29 | 6 | 10 | 19 | 39 | 14 |  |
| III | 18 (6.29%) | 3 | 1 | 2 | 0 | 0 | 5 | 1 | 0 | 3 | 3 | 0 |  |
| **TNM stage** |  |  |  |  |  |  |  |  |  |  |  |  |  |
| I—II | 164 (57.34%) | 13 | 36 | 30 | 8 | 5 | 19 | 2 | 6 | 16 | 20 | 9 |  |
| III—IV | 122 (42.66%) | 14 | 15 | 21 | 5 | 10 | 15 | 5 | 4 | 6 | 22 | 5 |  |
| **Serum CEA (ng/mL)** |  |  |  |  |  |  |  |  |  |  |  |  |  |
| < 5 | 172 (60.14%) | 21 | 30 | 31 | 9 | 10 | 24 | 4 | 6 | 12 | 17 | 8 |  |
| ≥5 | 109 (38.11%) | 4 | 21 | 19 | 4 | 5 | 10 | 3 | 4 | 9 | 24 | 6 |  |
| Unknown | 5 (1.75%) | 2 | 0 | 1 | 0 | 0 | 0 | 0 | 0 | 1 | 1 | 0 |  |

| **Table S2. Logistic regression analysis of association between SNPs and CRC risk** | | | | | |
| --- | --- | --- | --- | --- | --- |
| **Mutation**  **position** | **Mutant**  **type** | **Mutant number in CRC (n=286)** | **Mutant number in control (n=562)** | **Logistic regression** | |
|  |  |  |  | **OR（95%CI）** | ***P-value*** |
| 152 | T>C | 72 (25.17%) | 135 (24.02%) | 1.064 (0.763-1.476) | 0.712 |
| 199 | T>C | 17 (5.94%) | 65 (11.57%) | **0.483 (0.270-0.822)** | **0.010** |
| 235 | A>G | 30 (10.49%) | 47 (8.36%) | 1.284 (0.786-2.069) | 0.309 |
| 663 | A>G | 27 (9.44%) | 56 (9.96%) | 0.942 (0.574-1.513) | 0.808 |
| 709 | G>A | 72 (25.17%) | 110 (19.57%) | 1.382 (0.983-1.937) | 0.061 |
| 1736 | A>G | 27 (9.44%) | 49 (8.72%) | 1.091 (0.659-1.773) | 0.728 |
| 3010 | G>A | 45 (15.73%) | 92 (16.37%) | 0.954 (0.642-1.400) | 0.812 |
| 3970 | C>T | 42 (14.69%) | 80 (14.23%) | 1.037 (0.688-1.545) | 0.860 |
| 4071 | C>T | 15 (5.24%) | 50 (8.90%) | 0.567 (0.302-1.004) | 0.062 |
| 4248 | T>C | 28 (9.79%) | 55 (9.79%) | 1.000 (0.612-1.602) | 0.999 |
| 4715 | A>G | 35 (12.24%) | 59 (10.50%) | 1.189 (0.756-1.845) | 0.446 |
| 4824 | A>G | 27 (9.44%) | 58 (10.32%) | 0.906 (0.553-1.451) | 0.687 |
| 4883 | C>T | 51 (17.83%) | 126 (22.42%) | 0.751 (0.520-1.073) | 0.121 |
| 5231 | G>A | 22 (7.69%) | 29 (5.16%) | 1.532 (0.855-2.709) | 0.145 |
| 5417 | G>A | 23 (8.04%) | 33 (5.87%) | 1.402 (0.798-2.424) | 0.231 |
| 6392 | T>C | 42 (14.69%) | 79 (14.06%) | 1.052 (0.697-1.570) | 0.805 |
| 6455 | C>T | 15 (5.24%) | 49 (8.72%) | 0.579 (0.309-1.028) | 0.073 |
| 7853 | G>A | 15 (5.24%) | 40 (7.12%) | 0.722 (0.380-1.304) | 0.297 |
| 8414 | C>T | 41 (14.34%) | 89 (15.84%) | 0.889 (0.591-1.320) | 0.566 |
| 8584 | G>A | 51 (17.83%) | 88 (15.66%) | 1.169 (0.797-1.701) | 0.419 |
| 8701 | A>G | 138 (48.25%) | 289 (51.42%) | 0.881 (0.662-1.171) | 0.383 |
| 8794 | C>T | 27 (9.44%) | 54 (9.61%) | 0.981 (0.596-1.580) | 0.937 |
| 9540 | T>C | 137 (47.90%) | 293 (52.14%) | 0.844 (0.635-1.122) | 0.244 |
| 9950 | T>C | 21 (7.34%) | 30 (5.34%) | 1.405 (0.780-2.489) | 0.248 |
| 10398 | A>G | 157 (54.90%) | 331 (58.90%) | 0.849 (0.637-1.132) | 0.265 |
| 10400 | C>T | 137 (47.90%) | 285 (50.71%) | 0.894 (0.672-1.188) | 0.439 |
| 10609 | T>C | 27 (9.44%) | 44 (7.83%) | 1.227 (0.735-2.015) | 0.424 |
| 10873 | T>C | 137 (47.90%) | 288 (51.25%) | 0.875 (0.658-1.163) | 0.357 |
| 11914 | G>A | 22 (7.69%) | 42 (7.47%) | 1.032 (0.594-1.747) | 0.909 |
| 12372 | G>A | 21 (7.34%) | 34 (6.05%) | 1.231 (0.691-2.146) | 0.471 |
| 12406 | G>A | 27 (9.44%) | 48 (8.54%) | 1.116 (0.673-1.817) | 0.663 |
| 12705 | C>T | 189 (66.08%) | 376 (66.90%) | 0.964 (0.714-1.305) | 0.811 |
| 12882 | C>T | 27 (9.44%) | 47 (8.36%) | 1.142 (0.688-1.863) | 0.599 |
| 14569 | G>A | 16 (5.59%) | 37 (6.58%) | 0.841 (0.448-1.513) | 0.574 |
| 14668 | C>T | 41 (14.34%) | 90 (16.01%) | 0.878 (0.583-1.302) | 0.523 |
| 14783 | T>C | 137 (47.90%) | 332 (59.07%) | **0.637 (0.478-0.848)** | **0.002** |
| 15043 | G>A | 137 (47.90%) | 280 (49.82%) | 0.926 (0.696-1.231) | 0.597 |
| 15301 | G>A | 138 (48.25%) | 287 (51.07%) | 0.893 (0.672-1.188) | 0.438 |
| 16093 | T>C | 26 (9.09%) | 55 (9.79%) | 0.922 (0.557-1.49) | 0.745 |
| 16129 | G>A | 46 (16.08%) | 131 (23.31%) | **0.631 (0.432-0.908)** | **0.015** |
| 16140 | T>C | 23 (8.04%) | 38 (6.76%) | 1.206 (0.695-2.051) | 0.496 |
| 16172 | T>C | 23 (8.04%) | 42 (7.47%) | 1.083 (0.629-1.822) | 0.769 |
| 16189 | T>C | 95 (33.22%) | 166 (29.54%) | 1.187 (0.873-1.609) | 0.273 |
| 16217 | T>C | 32 (11.19%) | 55 (9.79%) | 1.161 (0.726-1.831) | 0.525 |
| 16223 | C>T | 184 (64.34%) | 368 (65.48%) | 0.951 (0.707-1.283) | 0.741 |
| 16234 | C>T | 16 (5.59%) | 31 (5.52%) | 1.015 (0.533-1.862) | 0.962 |
| 16261 | C>T | 33 (11.54%) | 54 (9.61%) | 1.227 (0.769-1.931) | 0.382 |
| 16298 | T>C | 39 (13.64%) | 79 (14.06%) | 0.965 (0.634-1.450) | 0.867 |
| 16304 | T>C | 36 (12.59%) | 80 (14.23%) | 0.868 (0.564-1.314) | 0.509 |
| 16311 | T>C | 43 (15.03%) | 165 (29.36%) | **0.426 (0.291-0.612)** | **<0.001** |
| 16319 | G>A | 43 (15.03%) | 85 (15.12%) | 0.993 (0.662-1.471) | 0.973 |
| 16362 | T>C | 94 (32.87%) | 228 (40.57%) | **0.717 (0.531-0.965)** | **0.029** |
| 5178A | C>A | 51 (17.83%) | 125 (22.24%) | 0.759 (0.525-1.084) | 0.135 |
| 1. The SNPs with frequency less than 5% or greater than 95% are not shown. | | | | | |

| **Table S3. Logistic regression analysis of association between SNPs and CRC risk** | | | | | |
| --- | --- | --- | --- | --- | --- |
| **Mutation**  **position** | **Mutant**  **type** | **Mutant number in CRC (n=286)** | **Mutant number in control (n=249)** | **Logistic regression** | |
|  |  |  |  | **OR（95%CI）** | ***P-value*** |
| 146 | T>C | 41 (14.34%) | 30 (12.05%) | 1.222 (0.740-2.038) | 0.437 |
| 150 | C>T | 58 (20.28%) | 57 (22.89%) | 0.857 (0.567-1.296) | 0.463 |
| 152 | T>C | 72 (25.17%) | 51 (20.48%) | 1.306 (0.871-1.970) | 0.199 |
| 195 | T>C | 30 (10.49%) | 19 (7.63%) | 1.419 (0.783-2.628) | 0.255 |
| 199 | T>C | 17 (5.94%) | 29 (11.65%) | **0.479 (0.252-0.886)** | **0.021** |
| 235 | A>G | 30 (10.49%) | 23 (9.24%) | 1.151 (0.652-2.058) | 0.629 |
| 489 | T>C | 137 (47.90%) | 122 (49.00%) | 0.957 (0.681-1.345) | 0.801 |
| 663 | A>G | 27 (9.44%) | 18 (7.23%) | 1.338 (0.723-2.530) | 0.359 |
| 709 | G>A | 72 (25.17%) | 51 (20.48%) | 1.306 (0.871-1.970) | 0.199 |
| 827 | A>G | 16 (5.59%) | 16 (6.43%) | 1.282 (0.580-2.893) | 0.536 |
| 1736 | A>G | 27 (9.44%) | 19 (7.63%) | 1.262 (0.687-2.360) | 0.457 |
| 3010 | G>A | 45 (15.73%) | 35 (14.06%) | 1.142 (0.709-1.851) | 0.587 |
| 3970 | C>T | 42 (14.69%) | 39 (15.66%) | 0.927 (0.577-1.491) | 0.753 |
| 4071 | C>T | 15 (5.24%) | 28 (11.24%) | **0.437 (0.222-0.827)** | **0.013** |
| 4248 | T>C | 28 (9.79%) | 21 (8.43%) | 1.178 (0.653-2.155) | 0.588 |
| 4715 | A>G | 35 (12.24%) | 21 (8.43%) | 1.514 (0.863-2.715) | 0.154 |
| 4824 | A>G | 27 (9.44%) | 21 (8.43%) | 1.132 (0.625-2.077) | 0.685 |
| 4883 | C>T | 51 (17.83%) | 48 (19.28%) | 0.909 (0.587-1.409) | 0.668 |
| 5231 | G>A | 22 (7.69%) | 17 (6.83%) | 1.137 (0.591-2.222) | 0.701 |
| 5417 | G>A | 23 (8.04%) | 15 (6.02%) | 1.364 (0.701-2.729) | 0.366 |
| 6392 | T>C | 42 (14.69%) | 37 (14.86%) | 0.986 (0.611-1.597) | 0.955 |
| 6455 | C>T | 15 (5.24%) | 28 (11.24%) | **0.437 (0.222-0.827)** | **0.013** |
| 6962 | G>A | 28 (9.79%) | 22 (8.84%) | 1.120 (0.625-2.030) | 0.705 |
| 7853 | G>A | 15 (5.24%) | 23 (9.24%) | 0.544 (0.272-1.058) | 0.077 |
| 8414 | C>T | 41 (14.34%) | 31 (12.45%) | 1.177 (0.715-1.954) | 0.524 |
| 8584 | G>A | 51 (17.83%) | 31 (12.45%) | 1.526 (0.947-2.495) | 0.086 |
| 8701 | A>G | 138 (48.25%) | 122 (49.00%) | 0.971 (0.691-1.364) | 0.864 |
| 8794 | C>T | 27 (9.44%) | 20 (8.03%) | 1.194 (0.654-2.210) | 0.566 |
| 9540 | T>C | 137 (47.90%) | 122 (49.00%) | 0.957 (0.681-1.345) | 0.801 |
| 9824 | T>C | 15 (5.24%) | 27 (10.84%) | **0.455 (0.231-0.865)** | **0.019** |
| 9950 | T>C | 21 (7.34%) | 13 (5.22%) | 1.439 (0.713-3.009) | 0.318 |
| 10310 | G>A | 42 (14.69%) | 38 (15.26%) | 0.956 (0.594-1.543) | 0.852 |
| 10398 | A>G | 157 (54.90%) | 138 (55.42%) | 0.979 (0.695-1.378) | 0.903 |
| 10400 | C>T | 137 (47.90%) | 121 (48.59%) | 0.973 (0.692-1.367) | 0.873 |
| 10609 | T>C | 27 (9.44%) | 21 (8.43%) | 1.132 (0.625-2.077) | 0.685 |
| 10873 | T>C | 137 (47.90%) | 109 (43.78%) | 1.181 (0.840-1.663) | 0.340 |
| 11914 | G>A | 22 (7.69%) | 16 (6.43%) | 1.214 (0.626-2.403) | 0.570 |
| 12358 | A>G | 20 (6.99%) | 19 (7.63%) | 0.910 (0.473-1.758) | 0.777 |
| 12372 | G>A | 21 (7.34%) | 19 (7.63%) | 0.959 (0.502-1.842) | 0.900 |
| 12406 | G>A | 27 (9.44%) | 21 (8.43%) | 1.132 (0.625-2.077) | 0.685 |
| 12705 | C>T | 189 (66.08%) | 156 (62.65%) | 1.162 (0.814-1.657) | 0.408 |
| 12882 | C>T | 27 (9.44%) | 21 (8.43%) | 1.132 (0.625-2.077) | 0.685 |
| 13759 | G>A | 15 (5.24%) | 16 (6.43%) | 0.806 (0.386-1.673) | 0.560 |
| 14569 | G>A | 16 (5.59%) | 15 (6.02%) | 0.924 (0.445-1.928) | 0.832 |
| 14668 | C>T | 41 (14.34%) | 31 (12.45%) | 1.177 (0.715-1.954) | 0.524 |
| 14783 | T>C | 137 (47.90%) | 122 (49.00%) | 0.957 (0.681-1.345) | 0.801 |
| 15043 | G>A | 137 (47.90%) | 122 (49.00%) | 0.957 (0.681-1.345) | 0.801 |
| 15301 | G>A | 138 (48.25%) | 124 (49.80%) | 0.940 (0.669-1.321) | 0.721 |
| 16129 | G>A | 46 (16.08%) | 48 (19.28%) | 0.803 (0.513-1.254) | 0.334 |
| 16140 | T>C | 23 (8.04%) | 15 (6.02%) | 1.364 (0.701-2.729) | 0.366 |
| 16172 | T>C | 23 (8.04%) | 33 (13.25%) | 0.572 (0.323-0.999) | 0.052 |
| 16189 | T>C | 95 (33.22%) | 77 (30.92%) | 1.111 (0.772-1.602) | 0.571 |
| 16217 | T>C | 32 (11.19%) | 29 (11.65%) | 0.956 (0.560-1.637) | 0.868 |
| 16223 | C>T | 184 (64.34%) | 153 (61.45%) | 1.132 (0.796-1.609) | 0.490 |
| 16261 | C>T | 33 (11.54%) | 25 (10.04%) | 1.169 (0.676-2.042) | 0.578 |
| 16274 | G>A | 17 (5.94%) | 13 (5.22%) | 1.147 (0.548-2.457) | 0.717 |
| 16290 | C>T | 28 (9.79%) | 23 (9.24%) | 1.066 (0.598-1.919) | 0.828 |
| 16298 | T>C | 39 (13.64%) | 26 (10.44%) | 1.354 (0.803-2.319) | 0.261 |
| 16304 | T>C | 36 (12.59%) | 35 (14.06%) | 0.880 (0.533-1.454) | 0.618 |
| 16311 | T>C | 43 (15.03%) | 40 (16.06%) | 0.925 (0.578-1.480) | 0.743 |
| 16319 | G>A | 43 (15.03%) | 30 (12.05%) | 1.292 (0.786-2.147) | 0.316 |
| 16362 | T>C | 94 (32.87%) | 86 (34.54%) | 0.928 (0.648-1.330) | 0.683 |
| 16519 | T>C | 152 (53.15%) | 135 (54.22%) | 0.958 (0.681-1.347) | 0.804 |
| 13928C | G>C | 42 (14.69%) | 42 (16.87%) | 0.848 (0.532-1.354) | 0.489 |
| 15487T | A>T | 34 (11.89%) | 20 (8.03%) | 1.545 (0.872-2.803) | 0.142 |
| 16182C | A>C | 21 (7.34%) | 15 (6.02%) | 1.565 (0.765-3.345) | 0.229 |
| 16183C | A>C | 51 (17.83%) | 39 (15.66%) | 1.169 (0.742-1.853) | 0.504 |
| 16257A | C>A | 18 (6.29%) | 14 (5.62%) | 1.127 (0.550-2.354) | 0.744 |
| 249d | A>D | 69 (24.13%) | 52 (20.88%) | 1.205 (0.802-1.818) | 0.372 |
| 315+C |  | 30 (10.49%) | 24 (9.64%) | 1.099 (0.625-1.949) | 0.745 |
| 5178A | C>A | 51 (17.83%) | 47 (18.88%) | 0.933 (0.601-1.449) | 0.756 |
| 7196A | C>A | 34 (11.89%) | 21 (8.43%) | 1.465 (0.832-2.633) | 0.191 |
| 1. The SNPs with frequency less than 5% or greater than 95% are not shown. | | | | | |

| **Table S4. Logistic regression analysis of association between SNPs and CRC risk** | | | | | |
| --- | --- | --- | --- | --- | --- |
| **Mutation**  **position** | **Mutant**  **type** | **Mutant number in CRC (n=286)** | **Mutant number in control (n=811)** | **Logistic regression** | |
|  |  |  |  | **OR（95%CI）** | **P-value** |
| 150 | C>T | 58 (20.28%) | 57 (7.03%) | **3.365 (2.267-4.998)** | **<0.001** |
| 152 | T>C | 72 (25.17%) | 186 (22.93%) | 1.131 (0.823-1.541) | 0.443 |
| 199 | T>C | 17 (5.94%) | 94 (11.59%) | **0.482 (0.273-0.802)** | **0.008** |
| 235 | A>G | 30 (10.49%) | 70 (8.63%) | 1.241(0.781-1.929) | 0.349 |
| 489 | T>C | 137 (47.90%) | 122 (15.04%) | **5.193 (3.846-7.032)** | **<0.001** |
| 663 | A>G | 27 (9.44%) | 74 (9.12%) | 1.038 (0.644-1.630) | 0.874 |
| 709 | G>A | 72 (25.17%) | 161 (19.85%) | 1.358 (0.985-1.861) | 0.059 |
| 1736 | A>G | 27 (9.44%) | 68 (8.38%) | 1.139 (0.703-1.798) | 0.585 |
| 3010 | G>A | 45 (15.73%) | 127 (15.66%) | 1.006 (0.689-1.447) | 0.976 |
| 3970 | C>T | 42 (14.69%) | 119 (14.67%) | 1.001 (0.678-1.454) | 0.996 |
| 4071 | C>T | 15 (5.24%) | 78 (9.62%) | **0.520 (0.284-0.894)** | **0.025** |
| 4248 | T>C | 28 (9.79%) | 76 (9.37%) | 1.050 (0.656-1.637) | 0.835 |
| 4715 | A>G | 35 (12.24%) | 80 (9.86%) | 1.274 (0.827-1.929) | 0.261 |
| 4824 | A>G | 27 (9.44%) | 79 (9.74%) | 0.966 (0.601-1.510) | 0.882 |
| 4883 | C>T | 51 (17.83%) | 174 (21.45%) | 0.794 (0.558-1.116) | 0.193 |
| 5231 | G>A | 22 (7.69%) | 46 (5.67%) | 1.386 (0.805-2.321) | 0.225 |
| 5417 | G>A | 23 (8.04%) | 48 (5.92%) | 1.390 (0.816-2.304) | 0.211 |
| 6392 | T>C | 42 (14.69%) | 116 (14.30) | 1.031 (0.698-1.500) | 0.874 |
| 6455 | C>T | 15 (5.24%) | 77 (9.49%) | **0.528 (0.288-0.907)** | **0.028** |
| 7853 | G>A | 15 (5.24%) | 63 (7.77%) | 0.657 (0.355-1.143) | 0.156 |
| 8414 | C>T | 41 (14.34%) | 120 (14.80%) | 0.964 (0.651-1.403) | 0.850 |
| 8584 | G>A | 51 (17.83%) | 119 (14.67%) | 1.262 (0.875-1.800) | 0.205 |
| 8701 | A>G | 138 (48.25%) | 411 (50.68%) | 0.907 (0.693-1.188) | 0.480 |
| 8794 | C>T | 27 (9.44%) | 74 (9.12%) | 1.038 (0.644-1.630) | 0.874 |
| 9540 | T>C | 137 (47.90%) | 415 (51.17%) | 0.877 (0.670-1.149) | 0.342 |
| 9950 | T>C | 21 (7.34%) | 43 (5.30%) | 1.415 (0.810-2.401) | 0.207 |
| 10398 | A>G | 157 (54.90%) | 469 (57.83%) | 0.887 (0.677-1.165) | 0.389 |
| 10400 | C>T | 137 (47.90%) | 406 (50.06%) | 0.917 (0.700-1.201) | 0.530 |
| 10609 | T>C | 27 (9.44%) | 65 (8.01%) | 1.196 (0.737-1.895) | 0.455 |
| 10873 | T>C | 137 (47.90%) | 397 (48.95%) | 0.959 (0.732-1.256) | 0.760 |
| 11914 | G>A | 22 (7.69%) | 58 (7.15%) | 1.082 (0.637-1.777) | 0.762 |
| 12372 | G>A | 21 (7.34%) | 53 (6.54%) | 1.133 (0.658-1.887) | 0.640 |
| 12406 | G>A | 27 (9.44%) | 69 (8.51%) | 1.121 (0.692-1.768) | 0.631 |
| 12705 | C>T | 189 (66.08%) | 532 (65.60%) | 1.022 (0.770-1.361) | 0.882 |
| 12882 | C>T | 27 (9.44%) | 68 (8.38%) | 1.139 (0.703-1.798) | 0.585 |
| 14569 | G>A | 16 (5.59%) | 52 (6.41%) | 0.865 (0.471-1.506) | 0.622 |
| 14668 | C>T | 41 (14.34%) | 121 (14.92%) | 0.954 (0.644-1.389) | 0.811 |
| 14783 | T>C | 137 (47.90%) | 454 (55.98%) | **0.723 (0.551-0.947)** | **0.018** |
| 15043 | G>A | 137 (47.90%) | 402 (49.57%) | 0.935 (0.714-1.225) | 0.628 |
| 15301 | G>A | 138 (48.25%) | 411 (50.68%) | 0.907 (0.693-1.188) | 0.480 |
| 16093 | T>C | 26 (9.09%) | 66 (8.14%) | 1.129 (0.691-1.795) | 0.617 |
| 16129 | G>A | 46 (16.08%) | 179 (22.07%) | **0.677 (0.470-0.959)** | **0.032** |
| 16140 | T>C | 23 (8.04%) | 53 (6.54%) | 1.251 (0.739-2.056) | 0.389 |
| 16172 | T>C | 23 (8.04%) | 75 (9.25%) | 0.858 (0.516-1.376) | 0.539 |
| 16189 | T>C | 95 (33.22%) | 243 (29.96%) | 1.163 (0.869-1.548) | 0.306 |
| 16217 | T>C | 32 (11.19%) | 84 (10.36%) | 1.090 (0.700-1.663) | 0.694 |
| 16223 | C>T | 184 (64.34%) | 521 (64.24%) | 1.004 (0.759-1.333) | 0.977 |
| 16234 | C>T | 16 (5.59%) | 41 (5.06%) | 1.113 (0.598-1.978) | 0.724 |
| 16261 | C>T | 33 (11.54%) | 79 (9.74%) | 1.209 (0.777-1.843) | 0.389 |
| 16298 | T>C | 39 (13.64%) | 105 (12.95%) | 1.062 (0.708-1.564) | 0.767 |
| 16304 | T>C | 36 (12.59%) | 115 (14.18%) | 0.872 (0.577-1.290) | 0.502 |
| 16311 | T>C | 43 (15.03%) | 205 (25.28%) | **0.523 (0.361-0.744)** | **<0.001** |
| 16319 | G>A | 43 (15.03%) | 115 (14.18%) | 1.071 (0.726-1.555) | 0.723 |
| 16362 | T>C | 94 (32.87%) | 314 (38.72%) | 0.775 (0.582-1.027) | 0.079 |
| 16519 | T>C | 152 (53.15%) | 135 (16.65%) | **5.680 (4.228-7.658)** | **<0.001** |
| 13928C | G>C | 42 (14.69%) | 42 (5.18%) | **3.152 (2.005-4.957)** | **<0.001** |
| 249d | A>D | 69 (24.13%) | 52 (6.41%) | **4.641 (3.148-6.882)** | **<0.001** |
| 5178A | C>A | 51 (17.83%) | 172 (21.21%) | 0.806 (0.566-1.133) | 0.223 |
| a. The SNPs with frequency less than 5% or greater than 95% are not shown. | | | | | |
